# A New Classification Framework to Understand Evolutionary Transitions in Individuality

**DOI:** 10.1002/bies.70098

**Published:** 2026-01-22

**Authors:** Saskia Wilmsen, Christian Kost

**Affiliations:** ^1^ Department of Ecology School of Biology/Chemistry Osnabrück University Osnabrück Germany

**Keywords:** biological units, evolutionary transitions in individuality, evolutionary unit, individuality, life cycles, organismality, physiological unit, symbiosis

## Abstract

Life on Earth has evolved as a series of evolutionary transitions, during which lower‐level units merged to form a new and more complex higher‐level entity. Besides few canonical examples, many life forms exist for which it remains unclear whether or not they are about to complete the transition. This paucity of mechanistic understanding is likely due to an overemphasis on few model systems and a lack of criteria to compare disparate biological units. Here, we aim at filling this gap by proposing a new framework to classify different forms of biological organization, which considers two fundamental aspects: (i) the physiological component and (ii) the evolutionary component. Categorizing different biological units according to whether and how these aspects are represented yields six types of structural organization. Our framework allows to compare different organizational forms, and, in this way, provide insight into the evolutionary processes giving rise to these arrangements.

## Graphical abstract

Classifying biological entities based on whether and how the two fundamental aspects physiological and evolutionary components are represented yields six types of structural organization. The resulting framework allows to compare different forms of organization, and, in this way, provides insight into the evolutionary processes giving rise to these arrangements.

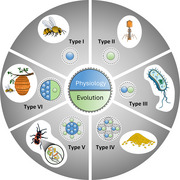

## 1 Introduction

Life on Earth has evolved in a series of evolutionary events, during which lower‐level units merged to form a new and more complex higher‐level entity [1, 2]. These evolutionary transitions radically transformed the structure and biology of the individuals involved. For example, once the transition has been completed, the initially independent units can only replicate as part of the newly formed larger whole, thus resulting in the formation of one coalesced evolutionary lineage [1, 2]. Moreover, after the transition, fitness is not a property of lower‐level units anymore, but has been exported to the higher‐level unit. This means that natural selection does no longer act on subunits individually. Instead, the newly formed higher‐level entity becomes the unit of selection [1, 3]. Due to these fundamental alterations, this process has been termed evolutionary transition in individuality (hereafter: ETI). Examples for ETIs include the formation of multicellular organisms from single cells, the emergence of eusocial insect societies from solitary insects, or the origin of eukaryotic cells from interactions between archaea and eubacteria [2, 4].

Two major evolutionary routes have been identified that can lead to ETIs. First, genetically unrelated and functionally different individuals can “come together” to form a higher‐level association [5], such as in aphids and their endosymbiotic *Buchnera* bacteria [6, 7, 8]. Second, genetically identical individuals can “stay together”, thus forming a larger, more complex structure [5], as has happened during the transition from unicellular to multicellular life.

However, changes in the organizational complexity of a biological entity do not always result in a fully completed ETI. Instead, many intermediate configurations exist, of which it is unclear whether or not they even have the potential to achieve this goal. For example, lower‐level units that make up a higher‐level individual are frequently not inherited together across generations (i.e., vertical transmission). Instead, interactions dissolve and are newly established in regular intervals (i.e., horizontal transmission), as for instance in microbial biofilms [9, 10]. In these cases, the lower‐level units involved do not form a joint evolutionary lineage that can be traced across generations.

Another important consequence that results from a nonpermanent association between lower‐level units is the inability of natural selection to operate on the composite assembly as a whole. Because lower‐level units strive to maximize their own fitness rather than the one of the higher‐level entity [11], conflicts of interest are expected to emerge that should favor competitive over cooperative interactions, thus hindering a complete functional integration of lower‐level units into a larger whole.

A major problem that arises from the abovementioned structural diversity is the difficulty to consistently classify different life forms according to a generally accepted nomenclature. To circumvent this issue, scientists have started to invent own categories to describe certain configurations such as the holobiont [12, 13], superorganism [14, 15], colonial organism [16, 17], or multispecies individual [8]. However, these categories are frequently applied exclusively to a relatively narrow set of taxonomic groups, thus limiting their usefulness.

The difficulty to name and consistently classify different kinds of structural organization is not just a semantic issue. In contrast, the study of ETIs would greatly benefit from the ability to qualitatively compare taxonomically different, yet structurally similar units. A universally applicable classification framework would therefore not only help to reveal similarities and differences between systems, but might also contribute to identifying criteria and conditions that drive ETIs. Thus, a unified classification framework is urgently required to consistently categorize life forms.

Here we aim at filling this gap by developing a new framework that allows to classify biological units with respect to ETIs. Our classification scheme is taxonomy‐independent and therefore applicable to a broad range of biological systems. Systematically assigning biological entities to clearly defined categories will help to understand the evolutionary trajectories giving rise to certain biological configurations and, in this way, may even provide mechanistic insights into the evolutionary transitions between them.

## 2 The Physiological and the Evolutionary Components Are Two Fundamental Aspects That Characterize Biological Entities

**FIGURE 1:**
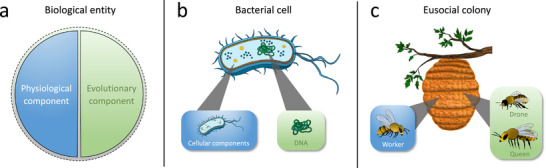
Biological units can be characterized by a physiological component and/or an evolutionary component. (a) The physiological component manifests as a coherent and integrated metabolic system, which defines the organismality of the focal entity. In contrast, the evolutionary component includes those aspects that allow the focal entity to evolve by natural selection, thus leading to the formation of traceable evolutionary lineages. (b) In a bacterial cell, both components are unified in a single individual: the physiological component is represented by most cellular components (blue), except the DNA, which serves as the evolutionary component (green). (c) In a eusocial insect colony, the two components are segregated into separate individuals that together form a higher‐level unit: Nonreproductive workers represent the physiological component (blue), and the queen and drones fill the role of the evolutionary component (green). Graphical representation of the biological unit modified after [21].

As a first step toward developing a framework to classify the structural organization of all existing forms of biological life, we reviewed the available literature to identify some of the main issues previous authors have encountered when describing biological units [18]. One of the key observations was that two different terms are frequently used: *organism* and *individual*. These terms are sometimes used interchangeably [19, 20] and have been subject to intense debate regarding their exact meaning [18, 19, 21, 22]. In addition, many authors apply them to rather specific types of biological units. In particular, the term *individual* is often used when referring to a spatially discrete unit that—as a whole—can participate in the process of natural selection [23]. In contrast, an *organism* is often viewed as a physiologically and metabolically integrated system that shows self‐preservation and is able to interact with its environment [21]. Accordingly, a given biological unit might meet the above definition of an organism without necessarily being classified as an individual. Take, for example, a mule that is clearly a living organism, but cannot reproduce and therefore does not evolve. Based on these considerations, we and others [21, 22, 23, 24, 25] have come to the conclusion that (i) the physiology and (ii) the ability to evolve are two independent properties that characterize biological units (Figure 1a).

In the following, we use the shortcut *physiological component* to collectively denote the organismic aspect of a given biological unit. In the context of our framework, we define the physiological component as a “self‐organized, physiologically integrated, and functionally autonomous biological system, which is spatially delimited and can consist of obligately interdependent subunits that contribute traits that are essential to ensure maintenance and growth of the superordinated entity” [18]. This includes metabolic functions such as the acquisition and processing of nutrients, regulation to ensure a functional integration of all constituent subunits, as well as means to interact with the biotic and abiotic environment [21, 22]. In addition, the physiological component provides for growth and maintenance of the superordinated entity, thus contributing vitally to an individual's evolutionary fitness. Importantly, the physiological component of a given biological unit is demarcated from the outside environment, thus defining the elements that are part of it and discriminating against those that are not (e.g., by the immune system or other self/nonself recognition systems).

In contrast, by *evolutionary component* we mean the ability of a focal biological unit to participate in the process of natural selection as a whole (Box 1). For this, a biological unit must differ phenotypically with regard to fitness‐relevant traits from other units, and this variation needs to be heritable (i.e., phenotypes of parents and offspring are correlated) [22, 26]. If these conditions are met, evolutionary change of biological units can be meaningfully represented in a phylogenetic tree, in which ancestors and descendants form one evolutionary lineage that can be traced across generations.

**Box 1 | Questions regarding the classification system**

In the following, some questions pertaining to our classification system are discussed with the aim to facilitate a better understanding of the overall framework.

**(1) Are the six types invariant categories?**

The main aim of the newly proposed classification system is to contribute to a better understanding of major evolutionary transitions in individuality (ETIs). Recognizing and distinguishing the physiological and the evolutionary component of a biological unit allows to categorize different forms of biological organization into clearly defined and distinct classes. These six types describe a specific configuration under certain ecological conditions. Since our classification system allows units to transition to or alternate between different types, depending on the developmental stage or environmental factors, the analysis of a given system is strongly context‐dependent. This flexibility is essential to also capture more dynamic systems that have been previously difficult to include into an overarching conceptual framework.

**(2) On which level should a system be analyzed?**

The new classification is primarily meant to serve as a heuristic tool that can help to conceptualize and contextualize certain configurations of biological organization. Most important in this context is the fact that the way a certain system is classified and subsequently analyzed strongly depends on the focal question. For example, a human being without its microbiome can be sensibly interpreted as a type III unit when the focus is on the higher level and not the constituent lower‐level units (e.g., mitochondria). Alternatively, the combination of a human as host organism and its microbiome can be classified as either type IV (mainly focusing on facultative bacterial symbionts) or type V (mainly focusing on obligate bacterial symbionts). Another example is an ant queen, which can be classified as a type II unit when it is considered as being part of the eusocial insect society (Figure 3d). However, the exact same individual can be viewed as a type III unit during its nuptial flight (Figure 3d). Shifting the focus according to needs allows ignoring components that appear irrelevant to a certain question, thus simplifying the overall analysis.

**(3) Can this classification system also be used to describe novel forms of organization that are currently not represented?**

Our classification system is primarily tailored to cases that are relevant in the context of ETIs. In this way, this conceptual framework can help to understand how the degree of cooperation and functional interdependence changes as biological units transition from one form of organization to another one. This is the reason why, in its current version, our framework focuses mainly on ecological interactions that are spatially associated and have a predominantly beneficial effect on the units involved.

However, this does not mean that the concept cannot be applied to configurations that are currently not included. For example, parasitic entities that obligately depend on the host for growth and survival, yet do evolve independently from the host's lineage (e.g., plasmid and bacterium, transmissible skin cancer and Tasmanian devil) can be classified as type II units when being considered in isolation, while they form a type V unit while being associated with their host.

Other ecological interactions, such as predator‐prey interactions, would be more difficult to include, since these interactions neither involve an enduring spatial aggregation of interaction partners nor do they benefit all participating units. However, in these cases, it also remains questionable how useful an analysis of these systems through the lens of the physiological/evolutionary component would be, since these ecological interactions are not known to represent evolutionary precursors of any of the ETIs known.

**(4) What are the similarities and differences between type III and type VI, and why is it possible in certain contexts to apply these categories interchangeably?**

The evolutionary pathways taken by known ETIs frequently resulted in an increase in hierarchical complexity. Translated into our classification framework, this involved a stepwise transition from a type III unit to a type VI unit. The final step in this sequence (type V to type VI transition) completes the coalescence of several previously independent units with the formation of a larger and more complex higher‐level entity. At this stage, the newly formed individual has become the relevant unit (e.g., fitness is now a property of the higher‐level unit). This means that the resultant type VI unit can be meaningfully interpreted as a type III unit if the constituent lower‐level units (e.g., single cells of multicellular organisms, mitochondria of a eukaryotic cell) are not immediately relevant to the focal question. However, depending on the organizational level that is of interest for a given analysis, the same type III individuals can also be interpreted as a type VI unit.

**(5) What exactly is meant by “interdependence”?**

Evolutionary transitions between different forms of biological organization frequently correlate with the evolution of a functional interdependence between lower‐level units. However, what exactly does *interdependence* mean? In the context of our framework, an interdependence between different biological units refers to a sharing of essential functions (i.e., division of labour) that allows the participating units to perform all physiological activities that are necessary for growth and survival. Functions fitting this definition include, for example, (i) a signalling molecule that a biological unit needs to induce a certain process, (ii) a growth‐essential metabolite, as well as (iii) the provisioning of services such as defence against predators or parasites. A hallmark of a functional interdependence is that the obligately dependent units typically lose their ability to exist in the absence of the other individuals, on whose functions they depend. Thus, viewed from the perspective of the higher‐level unit, interdependence causes emergent properties that the lower‐level units involved cannot produce in isolation.

**(6) Is the evolutionary component synonymous to the genetic or heritable component?**

The evolutionary component is not synonymous with the heritable unit, as the evolutionary component cannot be exclusively reduced to the genome of a biological unit. In contrast, it encompasses everything that is required by the focal unit to participate in the process of natural selection. The necessary characteristics include variation and inheritance of fitness‐relevant traits, which enable differential reproduction of biological units. While the underlying genetic constitution and the heritability of traits matter, the evolutionary components—as used in our framework—include more than these aspects.

An intuitive way to think about the distinction into evolutionary and physiological components is the segregation of functions in extreme forms of reproductive division of labour, as it is observed in eusocial insects or some multicellular organisms. Some cells/individuals are solely involved in sexual reproduction and thus represent the only contribution to the gene pool that is inherited across generations. They represent the evolutionary component, which includes—besides the genome—also certain phenotypic traits that are fitness‐relevant. The remaining cells/individuals make sure they allow these reproductive units to optimally fulfil this task to the best of their abilities. This includes providing energy or nutrients to reproductive individuals and protecting them from biotic and abiotic stressors. Thus, in these cases, these cells/individuals represent the physiological component. The fact that they also contain DNA and therefore could, at least theoretically, pass on their genes, does not matter, because in practice they do not. This is why the term evolutionary component includes all aspects of a biological unit that contribute to the formation of an evolutionary lineage.

Our approach particularly emphasizes the distinction between the physiological and the evolutionary component. Both components can be unified in a single individual (e.g., a bacterial cell, Figure 1b) or be segregated into separate individuals that together form a higher‐level unit (e.g., queen/drones and workers in a eusocial insect colony that shows reproductive division of labour, Figure 1c). The way in which the physiological and evolutionary components of subunits are organized likely depends on the preceding evolutionary history that gave rise to the corresponding structure.

## 3 All Biological Units Can Be Assigned to One of Six Categories

**FIGURE 2:**
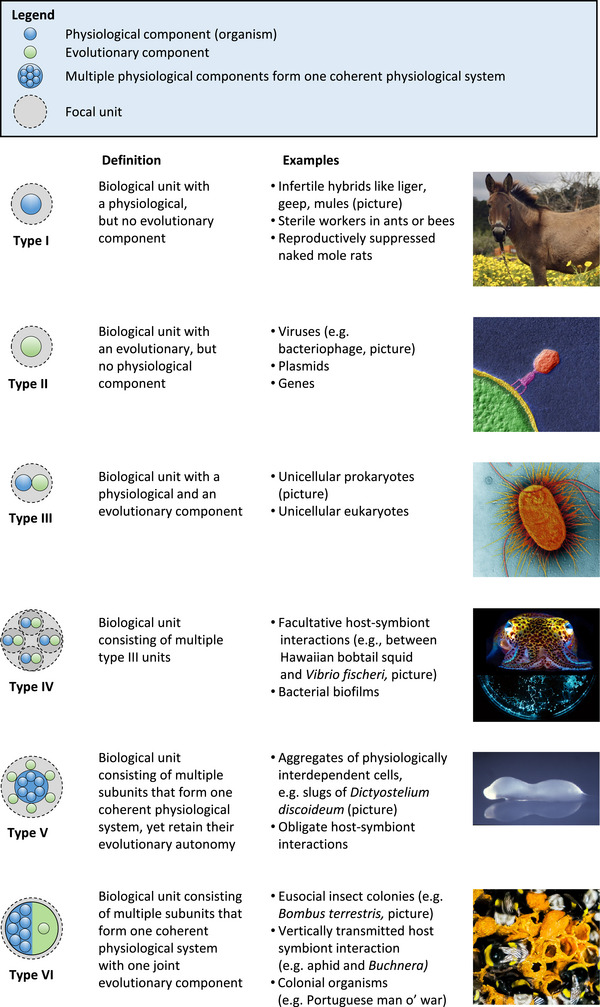
Structural organization of biological units. Systematically analyzing different biological units with regard to the structural implementation of the physiological and the evolutionary component(s) resulted in the identification of six main configurations (i.e., type I to type VI). Shown is a graphical representation of the main types (left), their definition, a list of examples, as well as an image of a representative example (right). Pictures show (from top to bottom) a mule (picture courtesy: Julia Krüger), a bacteriophage T4 (picture courtesy: eye of science, Meckes and Ottawa), a cell of *Escherichia coli* (picture courtesy: eye of science, Meckes and Ottawa), an Hawaiian bobtail squid *Euprymna scolopes* (picture courtesy: Mattias Ormestad), and bioluminescent *Vibrio fischeri* bacteria (picture courtesy: Marianne Engel), a slug of the slime mold *Dictyostelium discoideum* (picture courtesy: Louise Fets, Rob Kay, and Francisco Velazquez), and a eusocial insect colony (here: *Bombus terrestris*) (picture: Saskia Wilmsen).

After having established the physiological and evolutionary component as two distinct aspects that characterize biological entities (Figure 1), we analyzed different biological units to identify whether both elements are represented and if so, how they are structurally integrated into the focal unit. Our analysis revealed a total of six different types of biological units (Figure 2). These main categories differ in their structure, their degree of interdependence, as well as their level of heritability across generations. By *structure* we mean the extent, to which the focal unit consists of one or several subunits with independent or conjoined physiological and evolutionary components. In this context it is possible that the focal unit is characterized by only one of the two aspects. The *degree of interdependence* denotes the extent, to which subunits function as an integrated whole or rather as independent units (Box 1). Such a dependence can affect a unit's physiological component (e.g., inability to autonomously produce certain essential nutrients) and thus indirectly also its evolutionary component (e.g., a mutualistic fungus requires leaf‐cutting ants for dispersal). In the following, we will introduce the six main categories of biological units that emerge from our analysis of the existing variation in known biological systems.

### 3.1 Type I—Biological Unit With a Physiological, but No Evolutionary Component

Biological units of type I are characterized by an independently functioning physiological system and therefore classify as organisms. Due to their inability to produce fertile offspring, type I units do not form evolutionary lineages [22] (Figure 2). This circumstance can result from interspecific crossbreeding (e.g., mules) that yields sexually sterile individuals [22, 27, 28], reproductive division of labour (e.g., nonreproductive workers, Figure 1c), disease, physiological limitations, or genetic defects.

### 3.2 Type II—Biological Unit With an Evolutionary, but No Physiological Component

This category includes units that are unable to autonomously perform all physiological processes they require to reproduce, yet can form traceable evolutionary lineages (Figure 2). Thus, units belonging to this group essentially depend on other biological entities that provide them with required functional capabilities (e.g., metabolites, enzymatic activities, defence, or dispersal) [29]. Despite their obligate dependence on other biological units, type II individuals can evolve by natural selection. Examples for type II units include genes, viruses, or plasmids [22] (Figure 2).

### 3.3 Type III—Biological Unit With a Physiological and an Evolutionary Component

Biological units of type III include life forms that feature both a physiological and an evolutionary component (Figure 2) [22]. Type III units are therefore capable of autonomously performing all physiological functions that are needed to survive and reproduce. In addition, life forms falling into this category evolve by natural selection and thus form a traceable evolutionary lineage. This group of biological units covers a wide range of unicellular organisms, such as free‐living bacteria. In addition, depending on the focal perspective, also more complex biological systems can be categorized as type III (Box 1). For example, even if a unicellular eukaryote contains mitochondria, it can be sensibly categorized as a type III unit when lower levels are not relevant to the current research question.

### 3.4 Type IV—Biological Unit Consisting of Multiple Type III Units

The fourth category comprises biological entities that result from the spatial aggregation of independent biological units, which, however, maintain their physiological and evolutionary autonomy (Figure 2). This group includes a wide range of physical associations between lower‐level individuals that engage in different kinds of ecological interactions (e.g., mutualism, commensalism, etc.). Although being part of a type IV unit frequently benefits the constituents, the interactions are per definition nonobligate and therefore transient in nature. The lower‐level individuals that make up type IV units are able to autonomously survive outside the group and reproduce independently (e.g., many host‐associated microorganisms). Consequently, type IV units do not form one combined evolutionary lineage that can be traced across generations.

Type IV units can emerge in one of two ways [5]. First, by staying together of genetically related units or second, by a coming together of genetically different units. An example of type IV units that are formed by staying together are green algae of the genus *Tetrabaena*. Members of this genus form colonies that consist of four cells, which attach to each other via an extracellular matrix. Since these cells can also survive autonomously upon separation [30], the constituent cells represent independent type III units. Other examples for type IV entities that result from the coming together of genetically different units are different kinds of facultative symbioses, such as the association between the Hawaiian bobtail squid and the luminescent bacterium *Vibrio fischeri* [31] or microbial biofilms (Figure 2).

### 3.5 Type V—Biological Unit Consisting of Multiple Subunits That Form One Coherent Physiological System, yet Retain Their Evolutionary Autonomy

Biological type V units consist of multiple, physiologically interdependent subunits that retain their evolutionary individuality (Figure 2). Type V units can consist of individuals of the same (e.g., slugs of *Dictyostelium discoideum*) [32, 33] or different species (e.g., obligately interdependent host‐microbiota associations) [13, 34, 35]. Due to a synergistic complementation of tasks (e.g., metabolism) and an emergent regulation of these processes among subunits, the whole entity must be considered as one integrated physiological system. Type V units do not propagate by producing offspring on the level of the collective that preserves the genotypic composition of the parental consortium across generations (i.e., vertical transmission). Instead, phases of mixing occur regularly, during which the constituent subunits establish new associations with other individuals via horizontal transmission. Therefore, members of this category remain evolutionarily independent and do not form a conjoined evolutionary lineage.

A prominent example of a type V unit is the multicellular slug that is formed by free‐living amoebae of *D. discoideum* when nutrients are scarce [33, 36] (Figure 2). Slugs develop into fruiting bodies (i.e., stalk plus spores) that serve to disperse single‐celled amoebae [33, 37]. Type V units also include a wide variety of obligate symbiotic associations between eukaryotic hosts and (some of) their associated microbes [34, 38]. Although these systems show an obligate dependence of partners at the physiological level while associated, they exhibit independent inheritance when transmitted horizontally. Also, microbial aggregates, in which the constituent microorganisms engage in obligate metabolic interactions (e.g., among auxotrophic bacteria), fall into this category [39].

### 3.6 Type VI—Biological Unit Consisting of Multiple Subunits That Form One Coherent Physiological System With One Joint Evolutionary Component

This category encompasses biological entities, which consist of several interacting subunits that form one cohesive physiological system and share one unified evolutionary component (Figure 2). Units belonging to this category can make up larger structures, in which all lower‐level units are physically attached to each other (e.g., multicellular organisms, host‐endosymbiont interactions). Alternatively, type VI units can also form one entity in which subunits are able to move and operate freely (e.g., eusocial insect colonies). In both of these cases, subunits have reached a level of functional integration (i.e., regulation, metabolism, physiology, etc.) through which they operate as one tightly integrated unit. In addition, type VI units form one coherent evolutionary lineage. This can be achieved by a strictly vertical co‐transmission of interacting partners, as is the case in some of the closest symbiotic associations. Examples include the interaction between pea aphids and their endosymbiont *Buchnera aphidicola* [40, 41], between sharpshooter insects and their primary endosymbiont *Sulcia muelleri* [42], or in lichens, in which both fungi and algae are vertically inherited during vegetative reproduction [43]. Another mechanism of how a joint evolutionary component on the level of a type VI unit can be realized is by reproductive division of labour (e.g., monogynous queen/ drones in eusocial insect colonies or germline‐soma distinction in multicellular organisms).

Generally, type VI units require a more detailed explanation, because this kind of organizational structure marks the completion of a major evolutionary transition in individuality (Box 1). Both the physiological and the evolutionary components of type VI units are highly integrated. This pattern was likely caused by the collective inheritance of subunits across generations that increased interdependence among subunits. Once the previously autonomously replicating units have merged into one conjoint obligate physiological unit with shared vertical inheritance, a new higher‐level unit (type VI) has emerged. Due to the irreversible coalescence of subunits and the formation of one joint evolutionary lineage, these associations can alternatively be viewed as type III units (Box 1). In other words, as soon as the interconnectedness between subunits is irreversible, the previously autonomous individuals that make up the higher‐level unit have become less relevant.

## 4 Biological Units Display Life Cycles

**FIGURE 3:**
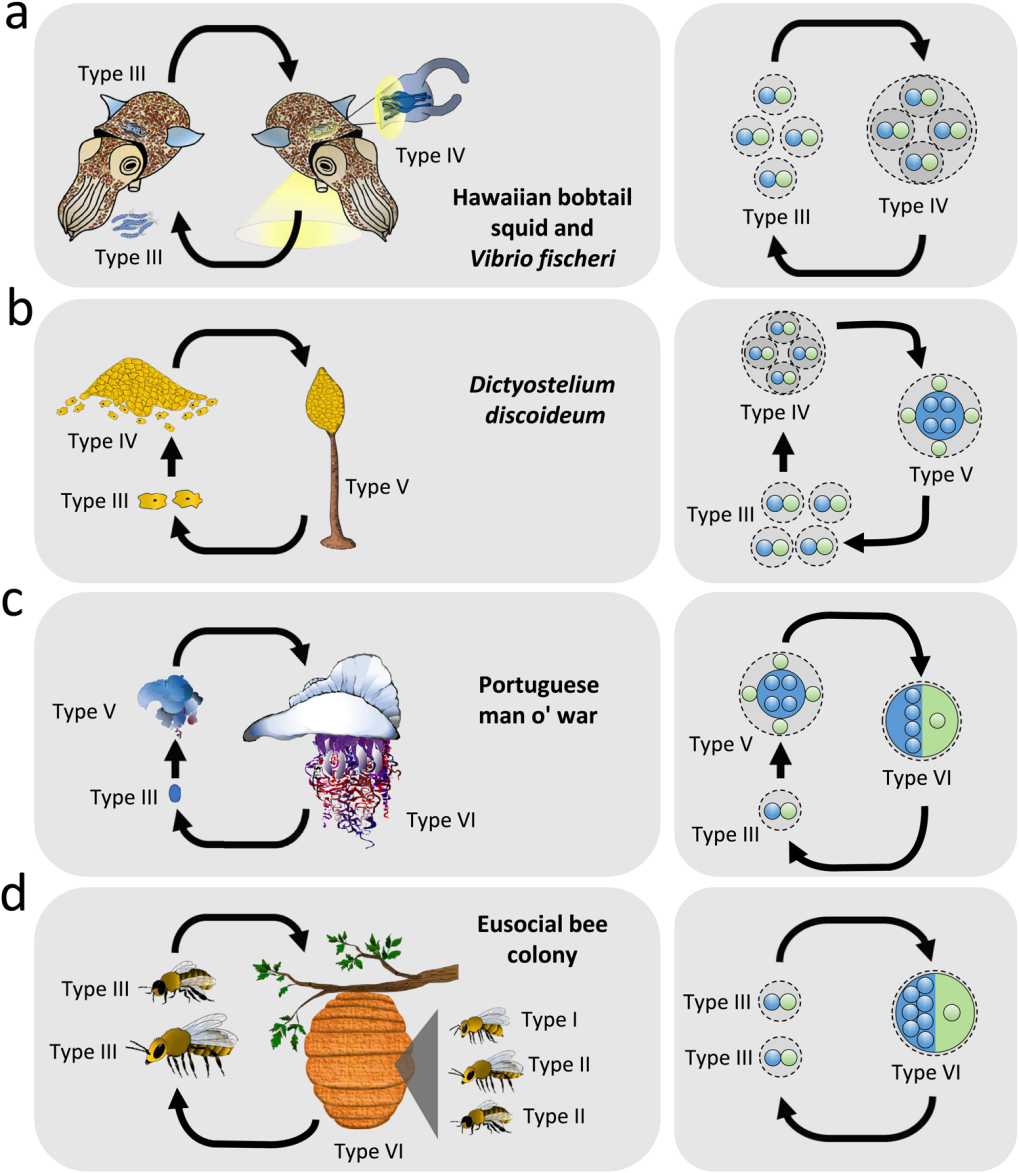
The assignment to categories can change with a individual's life cycle. Shown are life cycles of different biological units (left column) and the assignment of the corresponding life stages to the main categories (right column). (a) Symbiosis between Hawaiian bobtail squid (*E. scolopes*) and bioluminescent bacteria (*V. fischeri*) (i.e., transition from type III to type IV). (b) Life cycle of the slime mold *D. discoideum* includes the aggregation of individual amoebal cells into a slug, which then develops into a fruiting body (i.e., transition from type III over type IV to type V). (c) Portuguese man o’ war with the aggregation of zooids to a super‐ordinated colonial organism (i.e., transition from type III to type VI with an intermediate stage of type V). (d) A eusocial bee colony (e.g., honeybee *Apis mellifera*) collectively forms a highly integrated unit (type VI). It mainly consists of worker bees that represent physiological units (type I), because they are exclusively involved in maintaining the colony and ensuring homeostasis. In contrast, drones are categorized as reproductive units (type II), since they do not actively contribute to the maintenance of the colony, yet solely serve reproductive purposes. Outside the colony‐context, drones can be considered as type III units (Box 1). The same applies to the queen bee, who is the only unit within the nest to produce offspring, but does not actively participate in brood care (type II). Before the colony is founded, however, she represents a type III unit.

A striking observation that emerges, when different biological units are comparatively analyzed, is the fact that they all pass through different life stages (Figure 3). This includes besides ontogenetic changes of an individual's morphology, physiology, and behaviour, also transient associations with other biological individuals of the same or a different species. Even though the life stages of a given species can drastically differ from each other in terms of size and phenotypic capabilities, they are an inextricable component of the focal unit's biology.

Previously, life cycles have not been taken explicitly into account when thinking about the organismality or individuality of a given biological unit. This is reasonable for entities that spend the majority of their lifetime in a certain state and whose life cycle appears to be less important for the evolution of the whole unit. However, the situation is different for other life forms, in which life cycles and the associated dynamic changes represent a decisive factor (e.g., *D. discoideum*). In fact, life cycles have been identified to be of critical importance for the evolution of biological complexity [44]. Therefore, we think that a framework, which aims at classifying the structural organization of a given species, has to also take its life cycle into account.

Importantly, the two main components of biological units (i.e., physiological and evolutionary components) can undergo drastic changes as the focal unit transitions through different life stages. For example, within its life cycle, the Portuguese man o’ war passes through multiple stages that represent different categories of biological units (Figure 3c). A single polyp (type III unit) multiplies asexually through budding and produces additional type III units, which remain physically connected to each other. The resulting colony forms a coherent unit of a higher level, while its constituent subunits are functionally specialized [45, 46, 47, 48] (Figure 3c). None of the individual, genetically identical polyps are autonomously viable [47]. Thus, the newly formed superordinate unit represents one coherent physiological and evolutionary unit, which is consequently classified as a type VI unit in its mature state (Figure 3c).

Acknowledging that biological individuals are not just represented by their adult form, but frequently pass through various, structurally different life stages, allows not only to compare different taxonomic groups with regard to this variability, but also to potentially identify the evolutionary causes giving rise to the observed transitions. Another consequence of including life cycles into the perspective is the realization that different life stages are likely to also experience different selection pressures. Thus, the evolutionary trajectory of a certain biological entity is not only determined by one more or less arbitrarily chosen life stages, but also the result of natural selection operating on the individuals’ intermediate stages. Understanding this interplay can help explain why some groups of biological individuals transition to a new type of structural organization, while this pathway appears to be inaccessible to others.

## 5 The Classification of Biological Units Can Be Context‐Dependent

Biological units as well as ecological interactions [49] are frequently sensitive to environmental conditions. Moreover, environmental factors can induce the transition between different life stages [50] or determine the acceptance/ rejection of interaction partners by the immune system [21]. Thus, a system that aims at classifying biological individuals has to embrace context‐dependent variation (Box 1). For example, auxotrophic bacteria that are unable to autonomously produce certain metabolites can form multicellular clusters, within which all cells can grow due to the reciprocal exchange of the required compounds [51]. The resulting multicellular aggregate classifies as a type V unit. As soon as environmental levels of the required metabolites rise, clusters may fall apart, and cells can independently persist as type III individuals. Knowledge of whether the focal unit is sensitive to environmental changes is therefore critical when biological units are classified into different categories (Figure 2, Box 1). Moreover, understanding these changes can help to quantitatively predict the evolution of entities within the continuum of different types of biological individuals.

## 6 Experimental Approaches to Classify Biological Units

**FIGURE 4:**
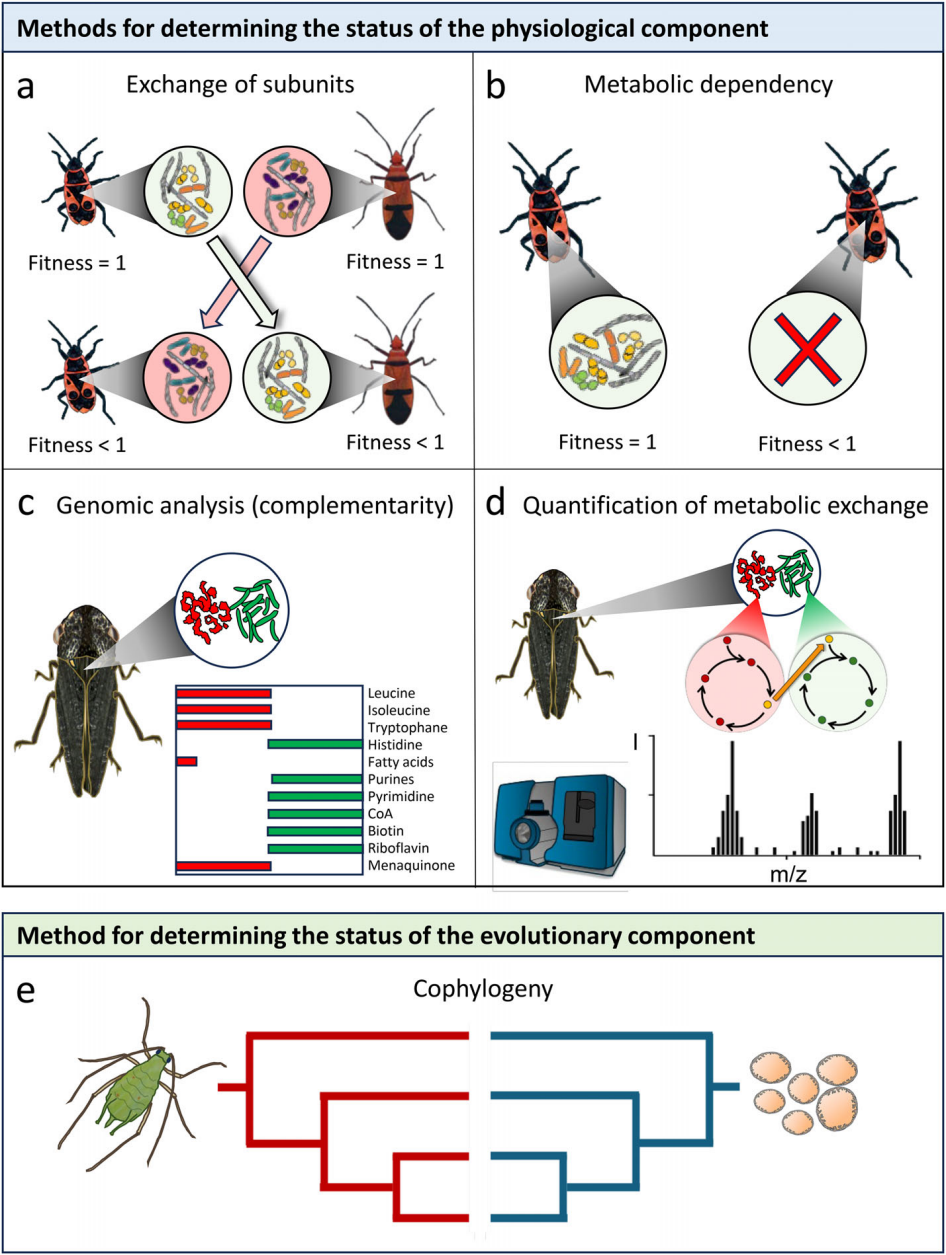
Experimental approaches to classify biological units by quantifying the status of the physiological (a–d) and the evolutionary component (f). (a) Reciprocal exchange of lower‐level units to determine the resulting fitness consequences. In the example, the microbiomes of two different host species (*Dysdercus fasciatus* and *Pyrrocoris apterus*) are reciprocally exchanged [52]. (b) Lower‐level units are removed to determine the degree of metabolic dependence between both parties. In this example, the microbiome of *P. apterus* is experimentally deleted [52]. (c) Genomic analyzes can identify metabolic complementarity in biosynthetic pathways among subunits. In the example, the genomes of two co‐occurring symbionts (*Sulcia muelleri* and *Baumannia cicadellinicola*) of the sharpshooter host are analyzed [53]. (d) Rates of metabolic exchange between subunits can be quantified by determining the flux of isotopically labeled metabolites using mass spectrometry [54]. The example shows a corresponding experiment in the *Sulcia*—*Baumannia* interaction [53]. (e) A congruent cophylogeny between host species and their symbiotic partners provides evidence for a strictly vertical transmission of the interaction [55]. The example shows the obligate endosymbiosis between the aphid *Acyrthosiphon pisum* and its intracellular bacterial symbiont *Buchnera aphidicola* [41, 56].

The applicability of a classification system heavily relies on our ability to unambiguously assign a given focal unit to one of the predefined categories. For this, diagnostic tests are essential that allow to verify some of the previously defined key characteristics in the focal unit (Figure 4). This is why, in the following, we suggest a set of experiments and analyzes that can help to elucidate the structural organization of a given biological unit.

Several tests can be used to probe the physiological component of a given biological unit. First, a powerful way to clarify whether subunits represent physiologically independent individuals is to analyze their specificity and interchangeability (Figure 4a). For example, replacing a bacterial symbiont of a host with other strains that either derive from another host or originate from an entirely different environmental source [52, 57, 58] allows to assess the degree of physiological dependence of the focal host: If the bacterial strain can easily be replaced by another one without significantly affecting host fitness, it would point to a nonspecific interaction and therefore suggest functional independence of the host. In contrast, observing a rather tight interaction, in which the bacterial strain cannot easily be replaced without negatively affecting host and symbiont fitness, indicates that the relationship is rather specific and thus more likely to represent one conjoint physiological component (i.e., type V unit). In this case, further experiments are required to demonstrate co‐transmission of host and symbiont across generations [59]. Another procedure to demonstrate metabolic interdependence is to analyze the performance (e.g., growth and survival) of subunits when they are part of the focal group as compared to their performance outside of it (Figure 4b). Their inability to survive outside their natural environment indicates an obligate dependence on other parties constituting the focal unit, thus limiting its independent existence. Modern technologies that analyze metabolic capabilities of subunits by comparatively analyzing their genomes or transcriptomes [60, 61, 62] (Figure 4c) or by quantifying rates of metabolic exchange by using isotopically‐labeled compounds [54] can provide even more detailed insights into the degree to which subunits form a coherent and functionally integrated unit (Figure 4d).

While the physiological component can be analyzed experimentally, probing the evolutionary component of a biological unit requires a reconstruction of its past evolutionary history. An effective way to achieve this is to create phylogenies of lower‐level constituents that comprise the focal unit (Figure 4e). If the resulting cophylogeny is characterized by highly congruent phylogenetic relationships [63], this finding would support the interpretation that all subunits form one inseparable evolutionary unit (i.e., type VI unit). In contrast, increasing degrees of incongruence of cophylogenies point toward a multipartite unit, whose components have retained their evolutionary individuality (i.e., type IV or V units).

Together, the above list of diagnostic tests and experimental designs, which, in many instances, have already been applied to various biological systems, can help to empirically elucidate the structural organization of a given biological unit.

## 7 Discussion

In this work, we proposed a new framework to classify and comparatively analyze biological units. First, our classification scheme distinguishes two main aspects of a given focal unit: (i) the physiological component and (ii) the evolutionary component (Figure 1a). Second, by qualitatively comparing different forms of organismal organization with regard to these two aspects, we identify six main categories that represent different configurations of how physiological and evolutionary components can be structurally implemented (Figure 2). Third, our conceptual framework recognizes life cycles as important components of the focal entities’ biology and acknowledges that the assignment to the six categories can change as focal units transition through their life cycle (Figure 3). Fourth, the categorization of biological units can depend on current environmental conditions and is therefore context‐dependent (Box 1). Finally, we suggest a set of diagnostic experiments that can be used to experimentally evaluate the current status of a biological entity's physiological and evolutionary components (Figure 4). Besides a semantic clarification, our framework also provides a new perspective on the existing diversity of structural arrangements that can be observed. The insights that are gained by analyzing these systems in the newly proposed way may help to resolve fundamental issues such as the conditions favoring evolutionary transitions between different forms of structural organization.

In our framework, classifying the status of a biological unit requires the inclusion of its life cycle. Multiple reason have motivated this decision. First, over their lifetime, individuals pass through a sequence of stages, which can differ dramatically in terms of their morphology, physiology, and ecology [64]. Consequently, during a life cycle, natural selection is likely to favor different features of different life stages [65]. This means that the combined outcome of these, possibly divergent selection pressures, needs to be considered in order to understand the evolution of certain structural configurations. Second, previous authors identified the emergence of a life cycle as a fundamental condition for an ETI to occur [44, 66]. This is mainly because higher‐level units need to reproduce in order to form the next generation of higher‐level units [66]. Thus, life cycles emerge automatically during the evolution of an increased structural complexity and are therefore intricately connected to the formation of a new, higher‐level individual [67]. Third, theoretical work suggests that once emerged, life cycles can (i) help to resolve conflicts between different levels of selection, (ii) cause the expression of heritable higher‐level traits, and (iii) allow selection to efficiently act on these emergent higher‐level traits [67]. Thus, detailed analyzes that compare different life stages of the same biological unit are required to fully understand its ETI. Fourth, increasing the structural complexity of a biological entity by merging lower‐level units will inevitably also cause trade‐offs within and between different levels of organization [68]. This is mainly because available resources need to be allocated to different processes, which, in the case of multipartite units, frequently correspond to different life stages (e.g., investment in growth of the higher‐level unit versus reproduction in the form of reproductive individuals) [68]. Consequently, the kind of life cycle a newly formed higher‐level entity uses to propagate will also affect its life history evolution. Including life‐cycles into the analysis can therefore help to understand the selection pressures that shaped the higher‐level entity. Even though the inclusion of life cycles appears to complicate our classification system at first sight, it also provides a fresh and, in our view, essential perspective on the evolutionary processes leading to the emergence of a new evolutionary individual as well as the physiological constraints and ecological factors shaping it.

A central question that needs to be addressed after categories of different biological entities have been defined is how representatives of these categories evolve and transition between different configurations over evolutionary time. One possibility is to comparatively analyze certain key parameters to identify mechanisms that may be causally involved in driving or hampering transitions between different types of biological units. Take, for example, the case of symbiosis, in which one or several symbionts live on or inside a host. This type of interaction includes a tremendous diversity of cases that drastically differ in both their form and function [69, 70]. The spectrum covered by symbiotic interactions ranges from (i) facultative associations, in which both parties can survive without their partner, yet benefit in its presence (i.e., type IV), over (ii) interactions, in which host and symbiont form an integrated physiological unit when interacting, yet are still able to change partners (i.e., type V), to (iii) more extreme cases, in which host and symbiont fuse to form one tightly integrated and inseparable unit (i.e., type VI). These categories represent different stages across a continuum, through which some host‐symbiont interactions pass over the course of their evolution. A factor that might be critical for governing these transitions is the mode, with which symbionts are transmitted between host generations [38, 59, 71]. While a strict vertical transmission has been suggested to be essential for the emergence of type VI units, high rates of mixing between genotypes (i.e., horizontally transmitted symbiotic interactions) may introduce genetic heterogeneity and thus thwart the alignment of evolutionary interests between host and symbiont [59, 72]. This example highlights the importance of considering life cycles for understanding evolutionary transitions in structural complexity.

A second possibility to analyze transitions between different types of biological units is to map how many cases can be found in either category and then compare the number of studies that report transitions between them. Such a meta‐analysis would reveal probabilities for transitions between different types of biological units and, in this way, also identify potential hurdles for the transition to occur. For example, if we assume that a strict vertical transmission of symbionts between host generations is essential for coupling the physiological and evolutionary unit of host and symbiont [59, 73, 74, 75, 76], both parties would have to find a solution that allows them to faithfully inherit the bacterial symbiont together with the host's germline. The likelihood, with which such an event occurs, would then determine the probability of a transition from type V to type VI. This example illustrates how a clearly defined conceptual framework can help to identify evolutionary mechanisms that are involved in causing major transitions in evolutionary individuality.

A third, very interesting observation that results from our work is that we did not find evidence for the existence of certain configurations that are theoretically conceivable. For example, we did not find examples of cases representing the functional mirror image of type V units (i.e., one conjoint evolutionary component with several independent physiological components). This finding is likely relevant because it suggests that a merging of the physiological unit may always precede the fusion of the evolutionary component. However, whether or not this is the case should be analyzed in future research.

Besides the new insights, our classification system potentially contributes to the analysis of major transitions in evolution; our framework can also inform research in other fields. For instance, when the evolution of certain biological units is analyzed across time, our model can help to predict coevolutionary change among the constituent lower‐level constituents [77]. For example, the evolution of the more integrated type VI unit should coincide with an increased number of regulatory pathways between subunits [78], enhanced self‐non‐self recognition systems [79], an increased level of functional complementarity [80], and a decreased level of functional redundancy [81]. These predictions can be logically deduced from theoretically analyzing different structural configurations and subsequently be tested experimentally. Another interesting research direction afforded by our classification system is to compare the life history evolution of different types of biological units (i.e., life stages) across different biological systems. For example, trade‐offs in resource allocation are likely to cause conflicts of interest among different life stages [82]. At the same time, over the course of an ETI, fitness is transferred from being a property of lower‐level units to becoming a trait of the newly formed higher‐level entity [83]. In this context, our classification framework can provide a heuristic tool to study interactions between different life stages as well as trade‐offs that might exist in some types of structural organization, but be broken during an evolutionary transition to another one [84].

A main advantage of a classification system, which strives at identifying structural similarities in divergent biological systems, is the new insights that can result when taking a different perspective. The power of this approach is beautifully showcased by previous work that has started to view eusocial insect colonies as “distributed organisms” [85]. This perspective matches our interpretation, in which both eusocial insect colonies and multicellular organisms are categorized as type VI units (Figure 2). Comparing these two cases reveals striking similarities, including, for example, the sequestration of a germline [85], propagation through a single cell bottleneck [85, 86], a self‐non‐self‐recognition system [79], communication among lower‐level units [78], and even apoptosis [87]. Furthermore, such comparative work has also identified kin selection as a major driver of both types of configurations [88] and ‐ in this way ‐ elucidated one of the key forces driving the evolution of organismal complexity. Thus, comparatively analyzing different taxonomic groups, which display some convergent evolutionary patterns, can help to identify shared mechanisms that have caused the evolution of the focal structures. Even more informative are possible differences that might be unearthed by this kind of analysis. Such differences, on for example a structural or physiological level, could help to provide causal explanations for the evolutionary trajectories taken by different life forms. Taken together, defining categories that are based on structural similarities can help to focus the view on similarities and differences in the mechanisms that caused the emergence and persistence of representatives of these categories.

## 8 Conclusion

A major question in evolutionary biology is how structural complexity evolves via the merging of previously independent biological units. In this work, we have provided a new classification system that aims at distinguishing different fundamental forms of structural organization that are likely relevant during an evolutionary transition in individuality. Several aspects that emerged from our analysis, such as (i) the distinction between the physiological and evolutionary unit [21, 22] or (ii) the importance of life cycles [65, 89, 90] are ideas that have been formulated previously. However, in our newly developed framework, we have connected and extended these thoughts. By defining the structural organization of a given biological unit, it is now possible to correlate and compare patterns that occur in seemingly incomparable biological entities. In this way, our classification system cannot only help to better understand evolutionary trajectories toward ETIs, but also inform research on the coevolution among interacting subunits and their life history evolution. With this work we hope to contribute to a resolution of disputes about semantics by providing a consistent language, thus refocussing the attention to what is most relevant—namely understanding how these different entities evolve. Future work should scrutinize the applicability of our classification system and, wherever necessary, refine or adjust it. Ultimately, we hope that the perspectives resulting from our integrative framework will stimulate further studies that aim at identifying the mechanisms that facilitate or hamper evolutionary transitions between different types of biological units.

## Author Contributions

S.W. and C.K. conceived and developed the presented concept. S.W. gathered and analyzed all data. S.W. and C.K. prepared all figures. S.W. and C.K. wrote the article.

## Conflicts of Interest

The authors declare no conflicts of interest.

## Data Availability

Data sharing is not applicable to this article as no datasets were generated or analyzed during the current study.
